# Garlic improves insulin sensitivity and associated metabolic syndromes in fructose fed rats

**DOI:** 10.1186/1743-7075-8-53

**Published:** 2011-07-27

**Authors:** Raju Padiya, Tarak N Khatua, Pankaj K Bagul, Madhusudana Kuncha, Sanjay K Banerjee

**Affiliations:** 1 Division of Pharmacology and Chemical Biology, Indian Institute of Chemical Technology (IICT), Hyderabad-500607, India

**Keywords:** Allium sativum L., garlic, Fructose, Diabetes, Metabolic syndrome, Nitric oxide, Hydrogen sulphide, oxidative stress

## Abstract

**Background:**

Type 2 diabetes mellitus, characterized by peripheral insulin resistance, is a major lifestyle disorder of the 21^st ^Century. Raw garlic homogenate has been reported to reduce plasma glucose levels in animal models of type 1 diabetes mellitus. However, no specific studies have been conducted to evaluate the effect of raw garlic on insulin resistance or type 2 diabetes mellitus. This study was designed to investigate the effect of raw garlic on fructose induced insulin resistance, associated metabolic syndrome and oxidative stress in diabetic rats.

**Methods:**

Male Sprague Dawley rats weighing 200-250 gm body weight were divided into 3 groups (n = 7 per group) and fed diet containing 65% cornstarch (Control group) and 65% fructose (Diabetic group) for 8 weeks. The third group (Dia+Garl group) was fed both 65% fructose and raw garlic homogenate (250 mg/kg/day) for 8 weeks. Whole garlic cloves were homogenized with water to make a fresh paste each day.

**Results:**

At the end of 8 weeks, serum glucose, insulin, triglyceride and uric acid levels, as well as insulin resistance, as measured by glucose tolerance test, were significantly (p < 0.01) increased in fructose fed rats (Diabetic group) when compared to the cornstarch fed (Control) rats. Administration of raw garlic to fructose fed rats (Dia+Garl group) significantly (p < 0.05) reduced serum glucose, insulin, triglyceride and uric acid levels, as well as insulin resistance when compared with fructose fed rats. Garlic also normalised the increased serum levels of nitric oxide (NO) and decreased levels of hydrogen sulphide (H_2_S) after fructose feeding. Although body weight gain and serum glycated haemoglobin levels of fructose fed rats (Diabetic group) were not significantly different from control rats, significant (p < 0.05) reduction of these parameters was observed in fructose fed rats after garlic administration (Dia+Garl group). Significant (p < 0.05) increase in TBARS and decrease in GSH was observed in diabetic liver. Catalase was not significantly affected in any of the groups. Administration of raw garlic homogenate normalised both hepatic TBARS and GSH levels.

**Conclusions:**

Our study demonstrates that raw garlic homogenate is effective in improving insulin sensitivity while attenuating metabolic syndrome and oxidative stress in fructose-fed rats.

## Background

Type 2 diabetes mellitus is the most common form of diabetes comprising 80% of all diabetic population. The World Health Organization has predicted that developing countries would have to bear the major burden of this disease. It has been estimated that there will be a 42% increase from 51 to 72 million individuals affected in the developed countries. In developing countries these figures are much higher and are expected to show 170% increase from 84 to 228 million [1,2]. The long term consequences of type 2 diabetes make it imperative to focus on the development of novel treatment strategies for the management of insulin resistance and the metabolic syndrome.

It is well known that dietary factors play a key role in the prevention of diabetes and other metabolic disorders [3-5]. Among all such agents, garlic has attracted the attention of modern medical science because of its widespread over the counter use. The salutary effects of garlic in type 1 diabetes are well established. Several studies document the efficacy of garlic in reducing blood glucose in various animal models of type 1 diabetes mellitus[6-10]. The hypoglycemic effect of garlic has been attributed to the presence of allicin and sulfur compounds [9]. Intraperitoneal injection of aqueous garlic extract has been reported to increase insulin sensitivity in rats administered low dose fructose orally. However, this parenteral route of administration of garlic does not mimic the effect of dietary garlic on diabetes. Moreover, in this model, only a slight (~8%) increase in blood glucose levels is observed while other metabolic changes associated with the metabolic syndrome have not been investigated [11].

Thus, the present study was designed to investigate the effect of dietary raw garlic homogenate on insulin resistance and the associated metabolic syndrome in an established model of type 2 diabetes mellitus characterized by insulin resistance, metabolic syndrome and oxidative stress [12-14].

## Methods

### *Preparation of garlic homogenate*

Fresh garlic (*Allium Sativum *L.) was purchased from a fixed shop in a local market in Hyderabad, India. Individual bulbs were put in a grinder to form a juicy paste as described earlier [15]. The garlic homogenate was prepared freshly each day.

### *Animals and treatment*

All animal experiments were undertaken with the approval of Institutional Animal Ethical Committee of Indian Institute of Chemical Technology, Hyderabad. Male Sprague-Dawley rats (200-250 gms) were purchased from the National Institute of Nutrition (NIN), Hyderabad, India. The animals were housed in BIOSAFE, an animal quarantine facility of the Indian Institute of Chemical Technology, Hyderabad, India. The animal house is maintained at temperature 22 ± 2°C with relative humidity of 50 ± 15% and 12 hour dark/light cycle. Animals were randomly divided into three groups (n = 7). Control group was fed 65% corn starch diet (Cat no. d11708b, Research diet, USA), whereas Diabetic group was fed 65% fructose diet (Cat no. d11707, Research diet, USA) for the induction of diabetes and associated metabolic disorders, [16] while the third group (Dia+Garl) was fed 65% fructose diet along with raw garlic homogenate (250 mg/kg) for a period of 8 weeks. Raw garlic homogenate was administered orally to rats using an oral gavage.

### *Biochemical assays*

Blood samples from all groups were analysed for various biochemical parameters at different time intervals to confirm the induction of diabetes and metabolic syndrome. Serum glucose and triglyceride levels were determined after 3 and 8 weeks of feeding, while serum insulin, uric acid, total cholesterol, nitric oxide and H_2_S were determined after 8 weeks of feeding. Glycated haemoglobin was determined from blood after 8 weeks of feeding. Blood was collected at 3 and 8 weeks from the retro-orbital plexus using small capillary tubes, centrifuged at 4000 rpm for 10 min. at 4°C, and serum was collected for all biochemical assays.

### *Estimation of glucose, uric acid, total cholesterol and triglyceride*

Serum samples were analysed for estimation of uric acid, total cholesterol and triglyceride levels using an auto blood analyser (Bayer Corp. USA). Triglyceride (Sensitivity: 10 mg/dl), uric acid (Sensitivity: 0.2 mg/dl), and total cholesterol (Sensitivity: 10 mg/dl) kits were obtained from Siemens, India. Blood glucose was measured using glucometer (One Touch Horizon, Singapore).

### *Estimation of nitric oxide (NO)*

Nitric oxide was determined by a commercially available kit (Assay design, USA). The sensitivity of the kit is 0.222 µmole/L. Assay is based on reduction of NO_3_^- ^into NO_2_^- ^using nitrate reductase. The azo dye is produced by diazotization of sulfanilic acid (Griss Reagent-1) with NO_2_^- ^and then subsequent coupling with N-(1-napthyl)-ethylene diamine (Griss Reagent-2). The azo dye was measured calorimetrically at 540 nm. Serum NO level was expressed as µmol/L.

### *Estimation of hydrogen sulphide (H_2_S)*

Serum H_2_S concentration was measured as described by Cai et. al, 2007 [17] after some modifications. Briefly, 0.1 ml serum was added into a test tube containing 0.125 ml 1% zinc acetate and 0.15 ml distilled water. Then 0.067 ml 20 mM N, N-dimethyl-phenylene diamine dihydrochloride in 7.2 M HCL was added. This was followed by addition of 0.067 ml 30 mM FeCl_3 _in 1.2 M HCL. The absorbance of resulting solution was measured with a spectrophotometer at a wavelength of 670 nm. The H_2_S concentration in a solution was calculated according to the calibration curve of sodium hydrogen sulphide (NaHS: 3.12-400µmol) and data were expressed as H_2_S concentration in µmol/L.

### *Estimation of glycated haemoglobin*

Glycated haemoglobin was estimated by using ion exchange micro-columns (Biosystem Ltd, Spain). Detection limit of the kit is lower than 4.3%. After preparing the hemolysate, where the labile fraction is eliminated, haemoglobin was retained by a cationic exchange resin. Haemoglobin A1c (HbA1c) was specifically eluted after washing away the haemoglobin A1a and A1b fractions, and was quantified by direct spectrophotometric reading at 415 nm.

### *Estimation of insulin*

Quantitative estimation of serum insulin was done by rat insulin ELISA kits (Mercodia, USA). The sensitivity of the kit is 0.025 µg/l. It is a solid phase two-site enzyme Immunoassay. It is based on the direct sandwich technique in which two monoclonal antibodies are directed against separate antigenic determinants on the insulin molecule. During incubation, insulin in the sample reacts with peroxidase-conjugated anti-insulin antibodies and anti-insulin antibodies bound to microtitration well. A simple washing step removes unbound enzyme loaded antibody. The bound conjugate was detected by reaction with 3, 3', 5, 5'-tetramethylbenzidine. The reaction was stopped by adding acid and read using a spectrophotometer at 450 nm.

### *Estimation of TBARS, Catalase and GSH*

At the end of 8 weeks, rats were sacrificed by cervical dislocation, and liver samples were collected and stored at -80°C. Liver samples from each rat were homogenized in freshly prepared phosphate buffer saline with 20 times dilution. Tissue homogenate was used for the estimation of TBARS. Remaining volume of homogenate was centrifugation at 5000 ×g for 15 min at 4°C. The supernatant was collected and used for the estimation of GSH, catalase and protein levels.

The extent of lipid peroxidation (TBARS) in liver was determined by measuring malondialdehyde content based on the reaction with thiobarbituric acid (TBA) [18]. Data were expressed as nmoles per gm liver weight using extinction co-efficient of 1.56 × 10^-5 ^M^-1 ^cm^-1^. Catalase activity was determined by measuring the decomposition of hydrogen peroxide at 240 nm [19]. Data was expressed as units per mg of protein. Tissue glutathione (GSH) content in liver homogenate was measured by biochemical assay using a dithionitrobenzoicacid (DTNB) method [20]. Data were expressed as µg per gm liver weight. Protein in supernatant was determined by Bradford method.

### *Intraperitoneal glucose tolerance test*

In a separate experiment, rats from all three groups were injected intraperitoneally with a freshly prepared glucose load of 2 gm/kg of body weight. Blood was collected from the retro-orbital plexus just before injecting the glucose load (0 min) and at 5, 30, 60 and 120 min for the estimation of blood glucose using glucometer (One Touch Horizon, Singapore).

### *Statistical analysis*

All values are expressed as mean ± SEM. Data were statistically analysed using one way ANOVA for multiple group comparison, followed by Student's unpaired 't' test for group wise comparison. Significance was set at p ≤ 0.05. Data were computed for statistical analysis by using Graph Pad Prism Software.

## Results

### *Body weight gain*

There was no significant difference in body weight gain between Control and Diabetic groups after 8 weeks of feeding. However, a significant (p < 0.05) decrease in body weight gain was observed in Dia+Garl group when compared to both Control and Diabetic groups (Table 1).

**Table 1:** Body weight gain after 3 and 8 weeks of fructose feeding

	Control	Diabetic	Dia + Garl
**Body weight gain after 3 weeks**	42.62 ± 14.70	45.54 ± 13.95	18.12 ± 7.51*^,^†
**Body weight gain after 8 weeks.**	63.84 ± 14.53	65.78 ± 24.65	35.66 ± 24.79*^,^†

*p < 0.05 vs Control group; †p < 0.05 vs Diabetic group

### *Glucose levels*

**Figure 1:**
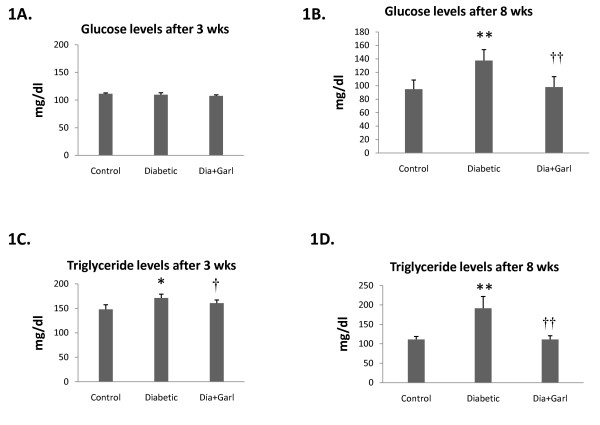
**Biochemical changes after administration of garlic in fructose fed rats**. Effect of garlic on blood glucose levels (A & B) and serum triglyceride levels (C & D) after 3 weeks and 8 weeks of fructose feeding. Data are shown as Mean ± SEM, *p ≤ 0.05, **p ≤ 0.01 vs Control group; †p ≤ 0.05, ††p ≤ 0.01 vs Diabetic group.

After 3 weeks of feeding, no significant change in blood glucose levels was observed in fructose fed rats (Diabetic group) compared to rats from Control group (Figure 1A). But after 8 weeks of feeding, rats from the Diabetic group showed a significant (p < 0.05) increase in blood glucose levels compared to Control rats. However, this increase in serum glucose levels in fructose feeding rats was significantly (p < 0.05) decreased after chronic administration of garlic (Dia+Garl group) (Figure 1B).

### *Triglyceride levels*

Serum triglyceride levels were measured at different time intervals during the study. A significant increase in serum triglyceride levels was observed after 3 and 8 weeks of fructose feeding in rats from Diabetic group. However this increased serum triglyceride level in fructose feeding rats was significantly (p < 0.05) decreased after chronic administration of garlic (Dia+Garl group) (Figure 1C &1D).

### *Serum insulin levels*

**Figure 2:**
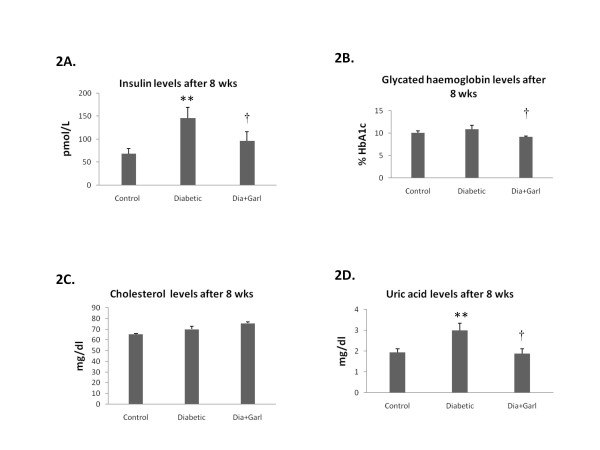
**Effect of garlic administration on serum insulin levels (A), glycated haemoglobin levels (B), cholesterol levels (C) and uric acid levels (D) after 8 weeks of fructose feeding**. Data are shown as Mean ± SEM, **p ≤0.01 vs Control group; †p ≤ 0.05 vs Diabetic group.

After 8 weeks, serum insulin levels were significantly (p ˂ 0.01) higher in the Diabetic group when compared to the Control group. Chronic administration of garlic (Dia+Garl group) significantly (p < 0.05) reduced serum insulin levels when compared to Diabetic group (Figure 2A).

### *Glycated Haemoglobin*

After 8 weeks, no significant increase in blood glycated haemoglobin levels was observed in Diabetic group as compared to Control. However, a significant (p < 0.05) decrease in blood glycated haemoglobin levels was observed in Dia+Garl group when compared to Diabetic group (Figure 2B).

### *Total cholesterol levels*

After 8 weeks, no significant change in serum cholesterol level was observed between Control and Diabetic group. Similarly no change in cholesterol levels was observed after chronic administration of garlic (Figure 2C).

### *Uric acid levels*

After 8 weeks, serum uric acid levels were significantly (p < 0.05) increased in the Diabetic group as compared to the Control group. Chronic administration of garlic (Dia+Garl group) significantly (p < 0.05) reduced serum uric acid levels as compared to Diabetic group (Figure 2D).

### *Nitric oxide levels*

**Figure 3:**
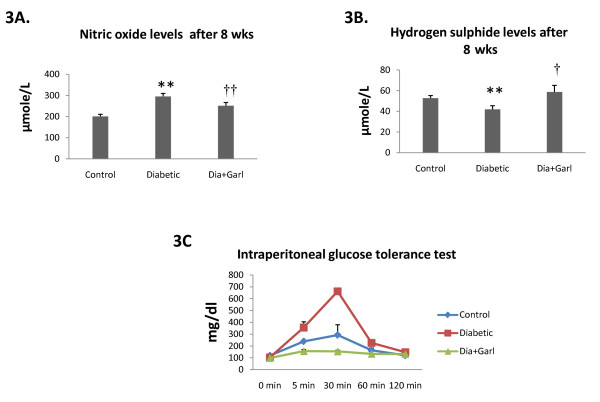
**Effect of garlic administration on serum nitric oxide levels(A) and serum hydrogen sulphide levels (B) after 8 weeks of fructose feeding.** (C) Effect of garlic administration on intraperitoneal glucose tolerance test. Data are shown as Mean ± SEM, **p ≤ 0.01 vs Control group; †p ≤ 0.05, ††p ≤ 0.01 vs Diabetic group.

Serum nitric oxide levels were significantly (p < 0.05) increased in the Diabetic group after 8 weeks as compared to the Control group. Chronic administration of garlic (Dia+Garl group) significantly (p < 0.05) reduced serum nitric oxide levels in fructose fed rats when compared to the Diabetic group (Figure 3A).

### *Hydrogen sulphide levels*

Serum hydrogen sulphide levels were significantly decreased (p < 0.05) in the Diabetic group after 8 weeks as compared to Control group. Chronic administration of garlic (Dia+Garl group) significantly (p < 0.05) increased serum hydrogen sulphide levels in fructose fed rats when compared to Diabetic group (Figure 3B).

### *Intraperitoneal glucose tolerance test*

An intraperitoneal glucose load led to a marked increase in blood glucose levels in Diabetic group, at 5 and 30 min, compared to the Control group. Chronic administration of garlic (Dia+Garl group) prevented this rise in serum glucose levels and was observed to be lower than the Control group (Figure 3C).

### *Hepatic TBARS, Catalase and GSH levels*

Hepatic TBARS levels were significantly increased (p < 0.05) in the Diabetic group after 8 weeks as compared to Control group. Chronic administration of garlic (Dia+Garl group) significantly (p < 0.05) decreased hepatic TBARS levels in fructose fed rats when compared to Diabetic group (Table 2). However, we did not observe any significant change in hepatic catalase activity in any of the groups (Table 2). Hepatic GSH levels were significantly decreased (p < 0.01) in the Diabetic group after 8 weeks as compared to Control group. Chronic administration of garlic (Dia+Garl group) significantly (p < 0.05) increased hepatic GSH levels in fructose fed rats when compared to Diabetic group (Table 2).

**Table 2:** TBARS, catalase and GSH levels in rat livers after 8 weeks

	Control	Diabetic	Dia + Garl
**TBARS (nmol/gm wet tissue)**	46.92 ± 5.70	60.51 ± 3.49*	47.22 ± 3.67†
**Catalase (U/mg protein)**	8.69 ± 1.7	10.88 ± 0.66	11.75 ± 1.25
**GSH (µg/gm wet tissue)**	176.95 ± 7.45	143.83 ± 3.73**	177.16 ± 16.72†

* p < 0.05 and ** p < 0.01 vs Control group; † p < 0.05 vs Diabetic group

## Discussion

High fructose corn syrup (HFCS) - a corn-based sweetener that has been on the market since 1970, is a popular food sweetener. Consumption of fructose in the form of HCFS is high in many countries including USA. Between 1970 and 1990, the consumption of HFCS increased over 1,000 percent [21]. High fructose intake over long periods is known to be hazardous for human beings as well as animals [21-23]. In the present study, a fructose rich diet was used for the induction of diabetes, which is characterized by insulin resistance and metabolic syndrome very much similar to human type 2 diabetes mellitus. Previous studies have shown that long-term fructose feeding induces diabetes associated with insulin resistance and metabolic syndrome in experimental animals such as rats and mice [16,24-27].

In the present study, rats were fed with a 65% fructose diet for a period of eight weeks in order to induce diabetes associated with insulin resistance and metabolic syndrome. Although blood triglyceride levels were increased after 3 weeks of high fructose feeding, we observed an increase in blood glucose level only after 8 weeks. Along with triglycerides, there was increase in other biochemical parameters associated with the metabolic syndrome such as uric acid and plasma insulin levels although blood cholesterol and glycated haemoglobin were not significantly affected in this rat model. Most importantly, insulin resistance, an important pathogenic mechanism in human type 2 diabetes and cause of all metabolic complications, was present in this model of diabetes, as evidenced by the altered glucose tolerance test.

Current medical research focuses on correcting insulin resistance, the primary underlying disorder in type 2 diabetes mellitus. Naturally occurring compounds represent a valuable source of such therapeutic agents of which garlic (*Allium sativum*) holds a unique position in history and is well recognized for its therapeutic potential for control of diabetes and its metabolic complications.

Although the antidiabetic effect of raw garlic has been well established in the type 1 experimental diabetic model [6-10] only one experimental study has been conducted so far to evaluate the effect of garlic on insulin resistance in rats [11]. However, in the study by Jalal et al [11] since low dose fructose was administered, only a marginal increase in blood glucose levels (~8%) was observed while the metabolic syndrome was not well characterized. Moreover, in this study, where an aqueous extract of garlic was administered intraperitoneally does not simulate the effect of oral intake of garlic. In the present study we evaluated whether oral administration of raw garlic homogenate improves insulin sensitivity and associated metabolic syndrome in fructose fed rats. The dose of 250 mg/kg was chosen as we have previously shown that garlic homogenate in this dose is effective in an animal model of heart disease and does not have any adverse effects [14,28].

In the present study, oral administration of raw garlic for a period of eight weeks showed salutary effects in an animal model of type 2 diabetes mellitus. There was significant reduction of blood glucose and improvement of insulin sensitivity in garlic treated rats. Other metabolic complications like increased serum triglyceride, insulin and uric acid levels observed in diabetic rats were also normalised after garlic administration. Lowering serum uric acid and triglyceride after garlic administration might be responsible for improving insulin resistance in fructose fed rats as there is evidence that fructose-induced insulin resistance is mediated by fructose-induced hyperuricemia or hypertriglyceridemia [24,25].

Increased weight gain and fat deposition is also responsible for insulin resistance [29]. However, we did not observe any increase of body weight gain and neither did the hepatic histopathological studies reveal any adiposity in fructose fed rats after 8 weeks (data not shown). After 20 weeks of high fructose feeding, Abdullah et al (2009) [30] did observed increased liver adiposity, but without any change in body weight. They also observed lipid deposition in liver section [30]. Hence it is likely that high fructose feeding in rats for 8 weeks is not enough to induce any adiposity and liver fat deposition.

An interesting observation of the present study is that chronic administration of garlic reduced body weight gain significantly compared to both control and diabetic rats. However, the reason for decrease body weight by garlic is not clear. Previously, it has been reported that allicin, one of the components of raw garlic paste, reduced weight gain in fructose fed rats [31,32]. The hypoglycaemic effect of garlic, has also been attributed primarily to the presence of allicin-type compounds [16,33,34]. Our finding on the significantly salutary effect of garlic on intraperitoneal glucose tolerance test is particularly promising and requires further elucidation. The insulin secretagogues activity of garlic and diallyl sulphide (active compound of garlic), may possibly contribute to this effect [35,36]. Reduction of body weight gain could also be responsible for improving insulin sensitivity in fructose fed rats.

NO and H_2_S are key players in disease progression [37-40]. Similar to NO, H_2_S is considered to be an important vasodilator, inducing endothelium-dependent and K^+^-ATP channel-dependent vasorelaxation in vivo and in vitro [41]. Increased serum levels of NO [42] and decreased levels of H_2_S [38] have been reported in diabetic patients. In the present study, we measured both serum NO and H_2_S levels in diabetic rats. Serum NO levels were significantly higher while H_2_S levels were significantly lower in diabetic rats compared to the control rats. Importantly, chronic administration of garlic normalised both gaseous molecules in fructose fed rats.

Increasing evidence in both experimental and clinical studies indicates that oxidative stress plays a major role in the pathogenesis of Type 2 diabetes mellitus. Free radicals are generated in diabetes by glucose oxidation. High levels of free radicals and the simultaneous decline of endogenous antioxidants can lead to damage of cellular organelles, and development of insulin resistance [41]. In the present study, high fructose feeding increased oxidative stress as evidenced by elevation of TBARS levels and reduction of GSH levels in diabetic liver in comparison to Control group. However, administration of raw garlic homogenate normalised both the increased TBARS and decreased GSH levels in diabetic liver.

Thus we may conclude that high fructose feeding for 8 weeks induces diabetes along with insulin resistance, metabolic disorder and oxidative stress. Oral administration of raw garlic homogenate increases insulin sensitivity and reduces metabolic complications along with oxidative stress in diabetic rats. Further human studies are essential to establish the role of garlic in controlling type 2 diabetes and its complications.

## Competing interests

The authors declare that they have no competing interests.

## Author details

RP (M.Sc) is Junior Research Fellow (ICMR), TNK (M. Pharm) is Senior Research Fellow (CSIR), PKB is doing MS in Pharmacology (NIPER, Hyderabad), MK (PhD) is Senior Technical Assistant and SKB (PhD) is Principle Investigator and Scientist in the Division of Pharmacology and Chemical Biology, Indian Institute of Chemical Technology (IICT), Hyderabad-500607, India.

## Authors' contributions

RP, TNK, PKB and MK carried out animal experimentation, biochemical estimation and statistical analysis of results. SKB conceived the study, and participated in its design, coordination and drafted the manuscript. The authors read and approved the manuscript.
